# The impact of compliance with 6-hour and 24-hour sepsis bundles on hospital mortality in patients with severe sepsis: a prospective observational study

**DOI:** 10.1186/cc3909

**Published:** 2005-11-11

**Authors:** Fang Gao, Teresa Melody, Darren F Daniels, Simon Giles, Samantha Fox

**Affiliations:** 1 Consultant, Critical Care Unit, Heart of England NHS Foundation Trust (Teaching), Birmingham Heartlands Hospital, Bordesley Green East, Birmingham B9 5SS, UK; 2 Research co-ordinator, Critical Care Unit, Heart of England NHS Foundation Trust (Teaching), Birmingham Heartlands Hospital, Bordesley Green East, Birmingham B9 5SS, UK; 3 Consultant, Critical Care Unit, Good Hope NHS Trust (Teaching), Rectory Road, Sutton Coldfield B75 7RR, UK; 4 Nursing consultant, Critical Care Unit, Heart of England NHS Foundation Trust (Teaching), Birmingham Heartlands Hospital, Bordesley Green East, Birmingham B9 5SS, UK; 5 Clinical nurse specialist, Critical Care Unit, Good Hope NHS Trust (Teaching), Rectory Road, Sutton Coldfield B75 7RR, UK

## Abstract

**Introduction:**

Compliance with the ventilator care bundle affects the rate of ventilator-associated pneumonia. It was not known, however, whether compliance with sepsis care bundles has an impact on outcome. The aims of the present study were to determine the rate of compliance with 6-hour and 24-hour sepsis bundles and to determine the impact of the compliance on hospital mortality in patients with severe sepsis or septic shock.

**Methods:**

We conducted a prospective observational study on 101 consecutive adult patients with severe sepsis or septic shock on medical or surgical wards, or in accident and emergency areas at two acute National Health Service Trust Teaching hospitals in England. The main outcome measures were: the rate of compliance with 6-hour and 24-hour sepsis care bundles adapted from the Surviving Sepsis Campaign guidelines on patients' clinical care; and the difference in hospital mortality between the compliant and the non-compliant groups.

**Results:**

The median age of the patients was 69 years (interquartile range 51 to 78), and 53% were male. The sources of infection were sought and confirmed in 87 of 101 patients. The chest was the most common source (50%), followed by the abdomen (22%). The rate of compliance with the 6-hour sepsis bundle was 52%. Compared with the compliant group, the non-compliant group had a more than twofold increase in hospital mortality (49% versus 23%, relative risk (RR) 2.12 (95% confidence interval (CI) 1.20 to 3.76), *P* = 0.01) despite similar age and severity of sepsis. Compliance with the 24-hour sepsis bundle was achieved in only 30% of eligible candidates (21/69). Hospital mortality was increased in the non-compliant group from 29% to 50%, with a 76% increase in risk for death, although the difference did not reach statistical significance (RR 1.76 (95% CI 0.84 to 3.64), *P* = 0.16).

**Conclusion:**

Non-compliance with the 6-hour sepsis bundle was associated with a more than twofold increase in hospital mortality. Non-compliance with the 24-hour sepsis bundle resulted in a 76% increase in risk for hospital death. All medical staff should practise these relatively simple, easy and cheap bundles within a strict timeframe to improve survival rates in patients with severe sepsis and septic shock.

## Introduction

Infection in hospitals continues to be a major concern for health boards and trusts throughout the UK and the rest of the world. Severe sepsis (infection-induced organ failure) usually develops as a consequence of infection in general medical and surgical wards, and is often initially managed by the non-intensive care medical team, although the patient's usual destination is an intensive care unit (ICU). Severe sepsis is common, the prevalence being approximately 2.26 cases per 100 hospital discharges and 68% of them require ICU care [1]. Severe sepsis is expensive; in the USA, the average cost per case is $22,100, with an annual total cost of $16.7 billion nationally. In the UK, although patients with severe sepsis represent 27% of ICU admissions, they account for 46% of all ICU bed days and 33% of all hospital bed days consumed by patients admitted to the ICU [2]. Costs are higher in non-survivors, ICU patients and patients with more organ failures [1]. Severe sepsis is frequently fatal, mortality rates remaining between 30% to 50% [3], or 500,000 deaths per year worldwide, with as many deaths annually as those from acute myocardial infarction and the number is projected to grow at a rate of 1.5% per year [4].

As the mortality rate of severe sepsis remains unacceptably high, a group of international critical care and infectious disease physicians, experts in the diagnosis and management of infection and sepsis, developed guidelines in 2004, termed the 'Surviving Sepsis Campaign (SSC) guidelines for management of severe sepsis and septic shock' [5].

A group of evidence based treatments related to a disease process, instituted together over a specific timeframe and termed 'a care bundle', is anticipated to result in better outcomes than when they are executed individually. For instance, the highest potential survival rate from cardiac arrest can only be achieved when the cardiac chain of survival, 'the care bundle' in cardiac arrest, occurs as rapidly as possible on site, and with each minute's delay the chances of a successful outcome fall by about 7% to 10% [6]. Compliance with the ventilator-associated pneumonia care bundle resulted in an average 44.5% reduction of ventilator-associated pneumonia [7]. The SSC group [4,8] has introduced the 'sepsis care bundle' into clinical practice with the goal of reducing mortality by 25% in five years. They recommend that individual hospitals codify the bundle elements, extracted from the SSC guidelines, into customised clinical protocols that function best in their institutions. We therefore instituted 6-hour and 24-hour sepsis bundles, modified from the SSC standard sepsis resuscitation bundle and sepsis management bundle, in our local hospitals.

There has so far been no information about compliance of sepsis bundles and its impact on outcomes. In this study, therefore, we determined the rate of compliance with 6-hour and 24-hour sepsis bundles and the impact of the compliance on hospital mortality in patients with severe sepsis or septic shock.

## Materials and methods

### Patient population

The study was conducted in two acute National Health Servie (NHS) Trust Teaching hospitals in England. The protocol was considered by the Local Research and Ethics Committee, and the need for informed consent was waived in view of the observational and anonymous nature of the study.

From 1 November 2004 to 31 March 2005, four authors (TM, DFD, SG and SF) ran daily screening on new admissions (aged 18 or over) into medical and surgical wards and accident and emergency areas for patients with severe sepsis or septic shock as defined by the International Sepsis Definitions Conference [9]. We then followed them up and used proximate look-back data extraction to record the time '0' when signs and symptoms of infection, documented source of infection and ≥1 organ disfunction had all been fulfilled.

### Six-hour basic ward care and six-hour sepsis bundle

All the patients were eligible for 6-hour basic ward care: such as oxygen, iv access and Modified Early Warning Scores (MEWS) [10] as well as the 6-hour sepsis bundle.

The elements of basic ward care and the 6-hour sepsis bundle adapted from the SSC standard sepsis resuscitation bundle are listed in Additional file 1. Our bundle differed from the sepsis resuscitation bundle as we used a haemoglobin target of 7 to 9 g/dl instead of haematocrit ≥ 30%, and used remaining hypotension after fluid resuscitation for threshold of inotropes instead of central venous oxygen saturation (ScVO2). A 'yes' score was obtained if the element had been executed, as documented on charts or notes, within the first six hours after time '0' (diagnosis of severe sepsis); a 'no' score was obtained otherwise.

### Twenty-four-hour sepsis bundle

If the process of severe sepsis was progressing and organ function support was required (for example vasopressors, mechanical ventilation), patients were reassessed for the appropriateness of critical care admission and of the 24-hour bundle; 69/71 patients were eligible to receive the 24-hour bundle as part of critical care management.

The elements of the 24-hour sepsis bundle adapted from the SSC sepsis management bundle is listed in Additional file 1. Again, a 'yes' score was obtained if the element had been executed, as documented on charts or notes, within the first 24 hours after time '0' (diagnosis of severe sepsis); a 'no' or 'not applicable' score was obtained otherwise.

### Definition of compliance

We assessed compliance using 'all or none' as a pass-fail basis for the whole bundle of elements. We used hospital death rate as the outcome measure.

### Statistical analysis

We applied Chi squared test, relative risks (RR) and their 95% confidence intervals (95%CI), and the number needed to treat (NNT), as appropriate, to compare hospital mortality between compliant and non-compliant groups.

## Results

### General information

**Figure 1:**
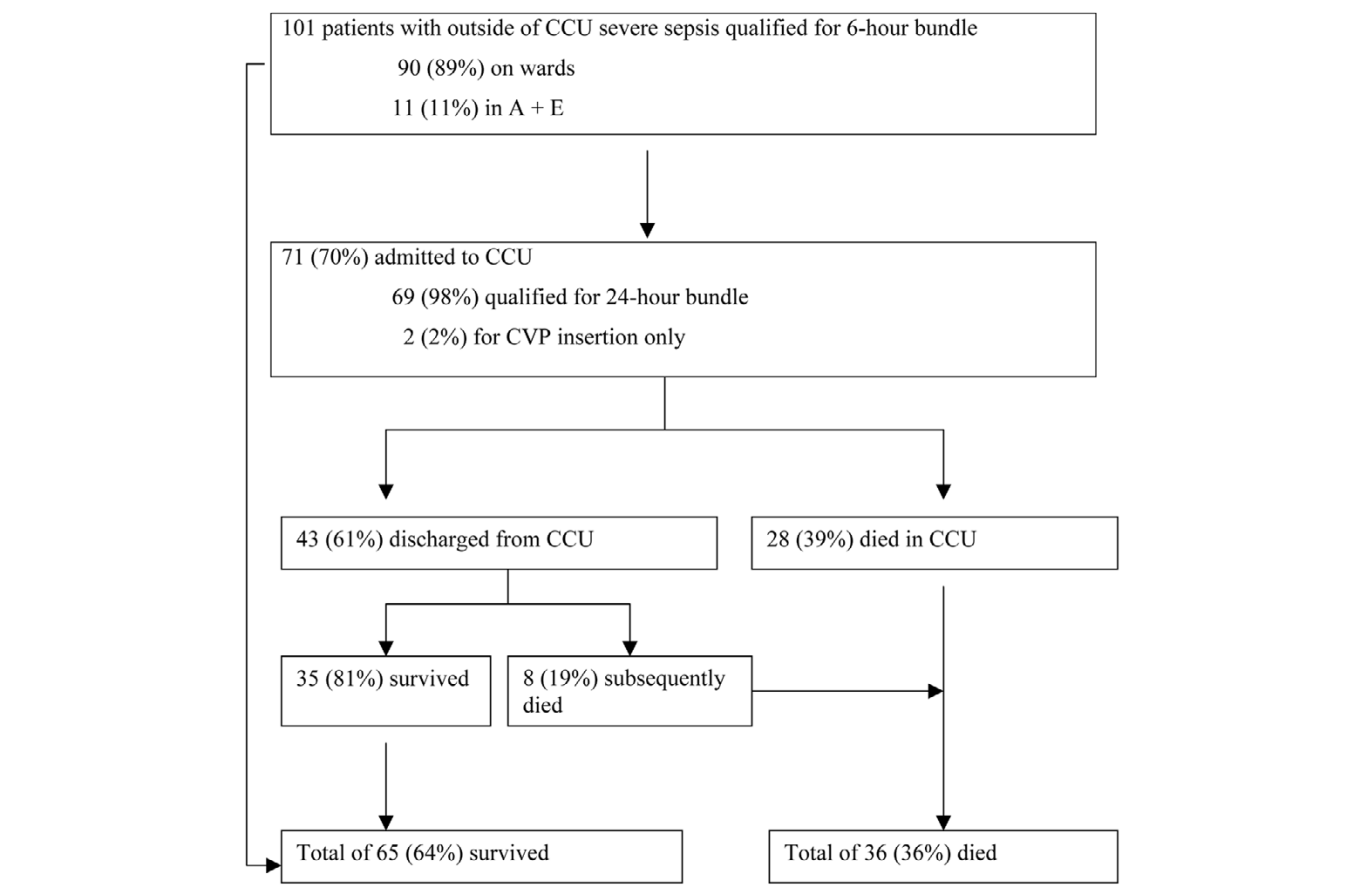
Patients flow chart. A + E, accident and emergency; CCU, critical care unit; CVP, central venous pressure.

We identified 101 consecutive patients who met inclusion criteria for severe sepsis or septic shock on the wards (n = 90), or in accident and emergency areas (n = 11). Of the 101, 71 (70%) were admitted into critical care units (high dependency unit, n = 20; ICU, n = 51) with a mortality rate of 39.4% (n = 28). The in-hospital mortality rate was 35.6% (n = 36). Figure 1 shows the patients' flow chart. General information about the patients is given in Table 1. The median age of the patients was 69 years (interquartile range 51 to 78), 53% were male and 56 (55%) were medical and 45 (45%) were surgical. The major sources of infection were chest (50%) and intra-abdomen (22%).

### Six-hour basic ward care

Within the first 6 hours following the diagnosis of severe sepsis, when patients had already developed one organ failure, we found that of the 101 patients, 8% had no oxygen administered, 14% had no iv access established, and 14% had no essential monitoring, including blood pressure, heart rate, respiratory rate, oxygen saturation, temperature, urine output and level of consciousness described as MEWS. One-third of the patients received no outreach service within the first 24 hours following the diagnosis of severe sepsis.

### Compliance with 6-hour sepsis bundle and hospital mortality

Of the 101 patients, within the first 6 hours after diagnosis of severe sepsis: 74% had a presumptive diagnosis made, including blood culture; 74% had antibiotics administered; 52% had serum lactate measured; in the event of hypotension, 84% had immediate 0.5 litre fluid administered; and in 70%, when MAP < 65 mmHg despite fluid resuscitation, a vasopressor was used and/or blood transfusion given to a haemoglobin target of 7 to 9 g/dl. All the elements of the first 6-hour sepsis bundle were received by 52% of patients and, by definition, the rate of compliance was 52%, with the lowest compliant element being the measurement of serum lactate.

Dividing the 101 patients into compliant (n = 52) and non-compliant (n = 49) 6-hour sepsis bundle groups, we found that the two groups were comparable in age, gender, sources of infection and type of specialties (Table 2). The two groups were not only comparable in their severity of sepsis at the points for interventions of the 6-hour sepsis bundle, assessed using median MEWS, but were also comparable for the appropriateness of further interventions, assessed using their requirement for the 24-hour sepsis bundle in critical care settings. Compared with the compliant group (Figure 1), however, we found that the non-compliant group had a more than twofold increase in hospital mortality (49% versus 23%, RR 2.12 (95% CI 1.20 to 3.76), *P* = 0.01). The number needed to treat to save one life was approximately 4.

Of the 101 patients, 71 (70%) were admitted into critical care and 59% (42/71) of these patients achieved all goals in the 6-hour sepsis bundle. The acute physiology and chronic health evaluation (APACHE) II score and the predicted hospital mortality were similar between the compliant and the non-compliant groups, although the hospital mortality was significantly higher in the non-compliant group (55% versus 29%, RR 1.93 (95% CI 1.08 to 3.45), *P* = 0.045) (Table 3). The number needed to treat remained approximately 4.

### Compliance with 24-hour sepsis bundle and hospital mortality

Of the 71 critical care patients, 2 (2%) required central venous pressure monitoring only prior to emergency laparotomy and wound debridement. Postoperatively, they were discharged to wards and required no further special care. Of 71 patients requiring organ support, 69 (98%) were qualified for the 24-hour sepsis bundle for clinical care. Of these 69 patients, within the first 24 hours following the diagnosis of severe sepsis: 64% received glucose control < 8.3 mmol/l; 43% had low-dose steroids given when requiring continued use of vasopressors; activated protein C was considered in only 30% of patients; and plateau pressures were maintained < 30 cm H_2_O in 85% of ventilated patients. The entire 24-hour sepsis bundle was achieved in only 30% of eligible candidates (21/69) and the rate of compliance, by definition, was 30%. Again, the compliant and the non-compliant groups were comparable in their characteristics and severity of sepsis, but hospital mortality was increased in the non-compliant group from 29% to 50% with a 76% increase in risk for death, although the difference did not reach statistical significance (RR 1.76 (95% CI 0.84 to 3.64), *P* = 0.16).

## Discussion

We found the rate of compliance with 6-hour and 24-hour sepsis bundles to be 52% and 30%, respectively. Patients with severe sepsis who did not receive the 6-hour sepsis bundle for their early management had a twofold increase in hospital mortality compared with the comparable group who were compliant with the bundle. Our low compliance is similar to other studies that reported initial low compliance following the publication of international guidelines, such as the management of ST segment elevation acute myocardial infarction (44%) or the management of stroke (26%) [11,12]. Our findings support previous studies showing that compliance with evidence-based guidelines significantly reduces mortality [13].

Informally, clinicians have used types of 'care bundles' since the early 1960s, when the pioneers of cardiopulmonary resuscitation established three components of cardiopulmonary resuscitation to be performed in unison in patients with ventricular fibrillation: closed chest cardiac massage, electrical defibrillation and artificial ventilation [14]. The theory of bundles in clinical care improvement, however, has only recently been developed [4]. Theoretically, the individual elements of a bundle are based on scientific evidence. Clinically, the aim of introducing bundles into clinical practice is primarily to improve process reliability, although the final endpoint in bundle development is to improve clinical outcomes.

In our study, each of the five interventions in the 6-hour sepsis bundle and each of the four interventions in the 24-hour sepsis bundle is backed by the SSC guidelines with grading recommendations of A to E. We chose, however, to deviate from the SSC bundle in our choice of the benchmark for persistent hypotension despite fluid resuscitation, adapting a target haemoglobin of 7 to 9 g/dl and/or vasopressors but excluding the requirement to achieve a target central venous pressure of >8 mmHg and ScVO2 of >70%. Both approaches are based on a grade B recommendation [15,16]. Both approaches aim to increase oxygen delivery to prevent or correct deficiency in oxygen delivery in severe sepsis either by a relatively programmatic and non-invasive approach (ours) or by a more scientific, invasive but more resource intensive approach (SSC). We felt this deviation from the SSC sepsis resuscitation bundle was necessary within our own trusts in the short term, due to resource limitation (ultrasound-guided access, training, staffing) preventing the safe and early placement of central venous catheters outside the critical care environment.

Our findings on inadequate ward care in critically ill patients replicate the old problem highlighted in a previous local report [17] and in the most recent National Confidential Enquiry into Patient Outcome and Death (NCEPOD) [18].

### Strengths and weaknesses of the study

The study has some notable strengths. To date, this is the first study to demonstrate the impact of compliance with an adaptation of the SSC 6-hour and 24-hour sepsis bundles on hospital mortality in patients with severe sepsis. Our findings add to the limited literature supporting the association between the use of a group of evidence-based interventions, executed together, and improved outcomes. In addition, the results suggest that if the association between use of process, indicated by compliance with evidence-based treatments, and improved mortality holds true, using process measures rather than the more resource-intensive outcome measures may be the better way for the NHS healthcare system to monitor performance and for the NHS hospitals to compare performance. Process measures based on the results of randomised controlled trials are able to detect relevant differences between hospitals that would not be identified by comparing hospital specific mortality, which is an insensitive indicator of the quality of care [19]. Finally, if the NNT is confirmed by future studies, sepsis care bundles will become the most powerful interventions in clinical care.

We recognise that the study also has some limitations. First, the nature of this observational study may have led to some unknown bias that, rather than interventions, may actually be the cause of both the differences in compliance with interventions and the differences in mortality observed. Second, we may not have measured the full clinical impact of these interventions. For example, we did not measure other risk factors for death, such as the severity of late stage of severe sepsis using a sequential organ failure assessment (SOFA) score, or patients' co-morbidity, which may have had an impact on decisions of withholding or withdrawal. Thirdly, we did not assess the patients (n = 30) who did not require critical care admission for inotropes, mechanical ventilation or drotrecogin alfa for glucose control. This approach also deviated from the SSC method. Finally, small sample size has resulted in the failure to demonstrate an association between compliance with the 24-hour sepsis bundle and hospital mortality.

## Conclusion

These pilot data suggest that compliance with 6-hour and 24-hour sepsis bundles can have a great impact on hospital mortality, although future studies will be needed to confirm these results. Efforts to improve hospital mortality from severe sepsis should focus on increasing compliance with these evidence-based interventions in appropriate patients.

## Key messages

• 	We instituted local 6-hour and 24-hour sepsis bundles, modified from the SSC standard sepsis resuscitation bundle and sepsis management bundle.

• 	For basic ward care, within the first 6 hours following the diagnosis of severe sepsis, when patients have already developed one organ failure, we found that 8% had no oxygen administered, 14% had no iv access established, and 14% had no essential monitoring, described as MEWS. One-third of the patients received no outreach service within the first 24 hours following the diagnosis.

• 	We found the rate of compliance with 6-hour and 24-hour sepsis bundles to be 52% and 30%, respectively. Compared with the compliant group with the 6-hour sepsis bundle for their early management, we found that the non-compliant group had a more than twofold increase in hospital mortality (49% versus 23%, RR 2.12 (95% CI 1.20 to 3.76), *P* = 0.01). The NNT to save one life was approximately 4.

• 	To date, this is the first study to demonstrate the impact of compliance with 6-hour and 24-hour sepsis bundles on hospital mortality in patients with severe sepsis. Our findings add to the limited literature supporting the association between the use of a group of evidence-based interventions, executed together, and improved outcomes.

• 	Future studies will be needed to confirm these results.

## Abbreviations

CI = confidence interval; ICU = intensive care unit; MEWS = Modified Early Warning Scores; NHS = National Health Service; NNT = number needed to treat; RR = relative risk; ScVO2 = central venous oxygen saturation; SSC = Surviving Sepsis Campaign.

## Competing interests

FG and TM were reimbursed by Critical Care Europe, Eli Lilly, for attending a seminar on Users of SSC Bundles, London.

## Authors' contributions

FG had the original idea, developed the design of the study, analysed the data and wrote the manuscript. TM, DFD, SG and SF contributed to the initial design, collected the data and helped interpret the results and revise the manuscript.

## Supplementary Material

Additional File 1 The sepsis care bundle audit form used listing the elements of basic ward care and the 6-hour and 24-hour sepsis bundles adapted from the SSC standard sepsis resuscitation bundle. Click here for file

## Figures and Tables

**Figure 2:**
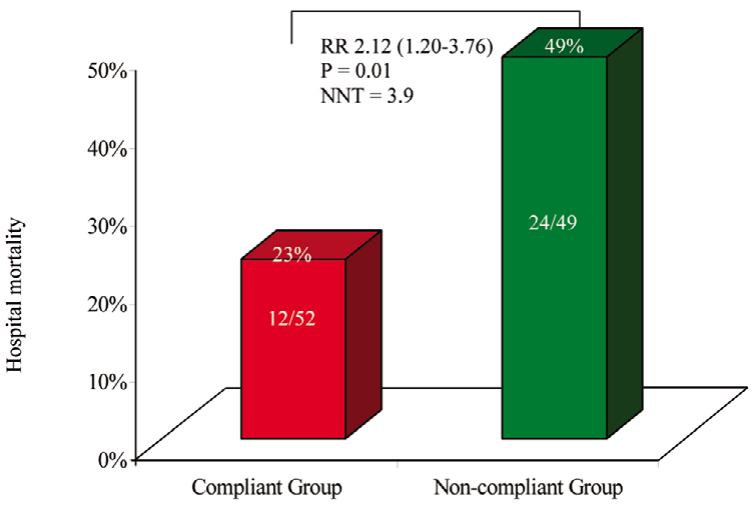
Compliance with the 6-hour sepsis bundle and hospital mortality (n = 101). NNT, number needed to treat; RR, relative risk.

**Table 1:** General patient information

Parameters	Results
Total number of patients	101
Age (years): median (IQR)	69 (51–78)
Gender: M/F (%)	53/48
Known cause of severe sepsis	87% (87/101)
Pneumonia	50% (50)
Intra-abdominal infection	22% (22)
Urinary tract infection	6% (6)
Others (skin, hip or CVP line)	9% (9)
Unknown causes of severe sepsis	13% (14/101)
Medical/surgical	55/45% (56/45)
Critical care admission	71% (71/101)
ICU mortality	39% (28/71)
Hospital mortality	36% (36/101)

CVP, central venous pressure; ICU, intensive care unit.

**Table 2:** Compliance with 6-hour sepsis care bundle and hospital mortality

Parameter	Patient groups (n = 101)		*P *value
	Compliant	Non-compliant	

N (%)	52 (52%)	49 (48%)	
Age, median (range)	69 (51–79)	69 (51–76)	NS
Sex (M/F; n)	28/24	26/23	NS
Pneumonia/abdomen (%)	56/26	55/25	NS
Medical/surgical (%)	57/44	56/45	NS
MEWS, median (IQR)	6 (5–6)	6 (5–6)	NS
Requiring 24-hour bundle	57% (39/69)	43% (30/69)	NS

IQR, interquartile range; MEWS, modified early warning scores; NS, no statistical significance.

**Table 3:** Compliance with 6-hour sepsis care bundle and hospital mortality in 71 patients admitted into critical care units

Parameter	CCU patient groups (n = 71)		*P *value
	Compliant	Non-compliant	

N (%)	42 (59%)	29	
Age, median (range)	71 (59–81)	70 (51–75)	NS
Sex (M/F; n)	22/20	17/12	NS
Pneumonia/abdomen (%)	55/25%	56/26%	NS
Medical/surgical (%)	55/45%	57/43%	NS
Requiring 24-hour bundle (%)	57% (24/42)	45% (13/29)	NS
MEWS, median (IQR)	6 (5–6)	6 (5–6)	NS
APACHE II, mean (SD)	19 (5.6)	20 (7.7)	NS
Predicted hospital mortality (n)	27% (16)	29% (19)	NS
Hospital mortality (n)	29% (12)	55% (16)	0.045^a^

^a^Relative risk 1.93, 95% confidence interval 1.08 to 3.45. APACHE, acute physiology and chronic health evaluation; CCU, critical care unit; IQR, interquartile range; MEWS, modified early warning scores; NS, no statistical significance; SD, standard deviation.
