# Supplementary material for: The impact of compliance with 6-hour and 24-hour sepsis bundles on hospital mortality in patients with severe sepsis: a prospective observational study
Source: Crit Care. 2005 Nov 11;9(6):R764–70. doi: 10.1186/cc3909 (PMC1414020; doi:10.1186/cc3909)
Supplement: Additional File 1 — The sepsis care bundle audit form used listing the elements of basic ward care and the 6-hour and 24-hour sepsis bundles adapted from the SSC standard sepsis resuscitation bundle. [file cc3909-S1.doc]

**Additional file 1**

Ref No: 12345………….

### Sepsis Care Bundle audit form

**Date:** / / **Gender:** M / F

**Date of Birth: Diagnosis made on Medical/Surgical Ward/ A&E area / HDU / ICU**

**Co-morbidity:**

**6 Hour Basic Ward Care**

- Oxygen **Yes / No**
- IV access **Yes / No**
- MEWS **Yes / No, value:**
- Outreach involved **Yes / No**
- Critical care admission **Yes / No**

**6 Hour Sepsis Bundle**

- Serum lactate measured **Yes /No, value:**
- Presumptive diagnosis **Yes / No, diagnosis:**
- Blood culture obtained, and then

antibiotics administered < 1 hr **Yes / No, name of antibiotics:**

- Immediate fluid 0.5 L/30 min **Yes / No**
- Vasopressors for MAP < 65 mmHg despite

fluid resuscitation **Yes / No**

- Inotropes and/or blood transfusion to target Hb 7-9g/dl **Yes / No**

**24 Hour Sepsis Bundle**

- Glucose control <8.3 mmol/L **Yes / No**
- Plateau pressure on average <30 cm H2O

for ventilated patients **Yes / No /n/a**

- Drotrecogin alfa considered for severe sepsis

using local guidelines **Yes / No**

- Steroids given for septic shock requiring continued

use of vasopressors **Yes / No /n/a**

- APACHE II **value**
- Predicted h-mortality % **value**

**Final outcome:**  Died in ICU **Yes / No**

Died in hospital **Yes / No**
